# Wearable Devices in Diving: Scoping Review

**DOI:** 10.2196/35727

**Published:** 2022-09-06

**Authors:** Benjamin Bube, Bruno Baruque Zanón, Ana María Lara Palma, Heinrich Klocke

**Affiliations:** 1 Faculty of Computer Science and Engineering Science University of Applied Sciences Cologne Gummersbach Germany; 2 Departamento de Ingeniera Informática Escuela Politecnica Superior Universidad de Burgos Burgos Spain; 3 Departamento de Ingeniería de Organización Industrial Escuela Politecnica Superior Universidad de Burgos Burgos Spain

**Keywords:** wearable device, underwater communication, head-up display, safety device, scuba diving, free diving

## Abstract

**Background:**

Wearables and their benefits for the safety and well-being of users have been widely studied and have had an enormous impact on the general development of these kinds of devices. Yet, the extent of research into the use and impact of wearable devices in the underwater environment is comparatively low. In the past 15 years, there has been an increased interest in research into wearables that are used underwater, as the use of such wearables has steadily grown over time. However, there has so far been no clear indication in the literature about the direction in which efforts for the design and construction of underwater wearable devices are developing. Therefore, the analysis presented in this scoping review establishes a good and powerful basis for the further development and orientation of current underwater wearables within the field.

**Objective:**

In this scoping review, we targeted wearable devices for underwater use to make a comprehensive map of their capabilities and features and discuss the general direction of the development of underwater wearables and the orientation of research into novel prototypes of these kinds of devices.

**Methods:**

In September 2021, we conducted an extensive search for existing literature on 4 databases and for grey literature to identify developed prototypes and early-stage products that were described and tested in water, could be worn and interacted with (eg, displays, buttons, etc), and were fully functional without external equipment. The studies were written in English, came from peer-reviewed academic sources, and were published between 2005 and 2021. We reviewed each title and abstract. The data extraction process was carried out by one author and verified by another author.

**Results:**

In total, 36 relevant studies were included. Among these, 4 different categories were identified; 18 studies dealt primarily with safety devices, 9 dealt with underwater communication devices, 7 dealt with head-up displays, and 2 dealt with underwater human-computer interaction approaches. Although the safety devices seemed to have gained the most interest at the time of this study, a clear trend toward underwater communication wearables was identified.

**Conclusions:**

This review sought to provide a first insight into the possibilities and challenges of the technologies that have been used in and for wearable devices that are meant for use in the underwater environment. Among these, underwater communication technologies have had the most significant influence on future developments. Moreover, a topic that has not received enough attention but should be further addressed is human-computer interaction. By developing underwater wearables that cover 2 or more of the technology categories that we identified, the extent of the benefits of such devices can be significantly increased in the future.

## Introduction

Over the past few years, wearables have been widely adopted and have become tools that many people use in their daily lives [1-3]. As a result, interest in using wearables for data collection and evaluation toward a scientific purpose has also increased in many areas within the last decade, especially for the monitoring of fitness and health-related metrics [4,5].

Divers can be divided into different categories, just like mainstream wearable users. These categories include scuba divers, who tend to be recreational divers, as well as free divers, who want to stay underwater for as long as possible with 1 breath. The transition from scuba diving to technical diving is fluent. We consider technical diving to be activities that are performed beyond the depths and conditions of scuba diving. Technical divers have a clear focus on performing professional activities underwater, which mostly involves dealing with the increased demands on the equipment and with the underwater conditions.

Although divers are generally at higher risk than nondivers, far fewer studies have been conducted in this area [6]. The underwater environment is, for humans, unnatural and dangerous, which makes it particularly necessary to survey physiological factors. Such factors that relate to the pulmonary, cardiovascular, neurological, and renal systems have so far been described in detail [7-9].

Due to the significantly smaller number of people who are divers, as well as the higher demands on wearable devices in terms of water resistance and water pressure, the development of underwater wearables has been challenging. As a result of the increased pressure under water, many sensors and actuators must be treated differently than they are on land. In particular, those that provide vital sign data, such as oxygen saturation monitors or heartbeat monitors (eg, Holter monitors), must be adapted to the different underwater conditions to function smoothly. Furthermore, water represents an almost impenetrable barrier for various radio networks, such as wireless local area networks or Bluetooth networks, and the propagation of radio waves under water decreases as the frequency increases. This results in enormous hurdles, especially when networking different wearables underwater, since radio wave–based connection methods cannot be used. In addition to wired connections, acoustic and optical data transmission have primarily been investigated and recognized as useful so far [10-12].

Ongoing development and research have made it possible to propose initial prototypes, concepts, and ideas in the field of diving physiology, and wearable sensors have also been extensively investigated recently [13,14]. Therefore, using devices to collect and process diving physiology data could be helpful in minimizing underwater dangers, such as drowning, the risk of floating away, or fear. Previous studies have already been able to collect and describe in detail the individual sensors that have been used underwater [13-15]. However, none of these studies went into more detail with regard to whether and how these sensors can be combined in a portable; compact; and, if possible, networked end device. In addition, only sensors that directly relate to the health of divers have been covered in the literature so far.

To close this gap and to show a first look at the tendencies of different wearables for any kind of diver and, therefore, for general underwater use, we especially tried to answer the following questions:

- What use cases do wearables cover, besides a depth logger dive computer, and in which directions are they developing?
- Which communication technologies and seals are the most forward-looking for wearables and up to what depth have they been used and tested?
- To what extent have wearable devices been tested and what results have been attained?
- Are there important topics that have only been covered to a very small extent in scientific literature?

For the sake of completeness, individual wearables from other reviews were included, provided that they were intelligent electronic devices that could be worn on the body or on the surface of the skin and could detect, analyze, or transmit information. Dive computers that use the classic approach of a pure depth sensor were not included in this review, as we considered them to be too simple to be compared with the devices that we defined as *wearables*. Similarly, commercial dive computers were not considered in this review for two reasons. First, the details of the specifications of individual dive computers can only be compared with great effort and not in a review, since they are often tested under manufacturer-specific conditions. Second, built-in sensors and actuators are often not disclosed and would thus have required a direct comparison in a laboratory. Purely commercial studies on dive computers have been published [16-19].

## Methods

The methodology used in this study was based on the approach of scoping reviews. The scoping review approach aims to present certain key concepts that have not or have only partially been reviewed so far.

In this review, we targeted wearable devices for underwater use to make a comprehensive map of their capabilities and features and discuss the general direction of the development of underwater wearables and the orientation of research and prototype designs for these kinds of devices.

For this study, a scoping review was conducted to identify and discuss the extent, scope, and nature of underwater wearable research; propose a summary of existing research; and identify gaps in the existing literature [20-22]. During the review process, we followed the PRISMA (Preferred Reporting Items for Systematic Reviews and Meta-Analyses) guidelines and the PRISMA-ScR (Preferred Reporting Items for Systematic Reviews and Meta-Analyses extension for Scoping Reviews) guidelines [23].

The search syntax was developed on PubMed and the Scopus database, using different word variations and combinations for the search in the “Title-Abstract” search field on PubMed and the “Article title, Abstract, Keywords” field on the Scopus database. Each iteration of the search results was searched for 10 publications that were previously known to the authors to randomly examine the results of the search. If all 10 papers were not found, the search was repeated with a different search string. The final search formula—“wearable OR device AND (diver OR diving)”—yielded the most complete and strongest results, which contained all 10 papers. Other terms did not yield any useful results in either database and were discarded. By using the final search string, which was created to be as generic as possible, many articles that were considered for inclusion in this study were found.

The final search was conducted on September 2021 within the PubMed, Scopus, and ACM databases, and we checked Google Scholar for additional literature that may have not been covered by any of the aforementioned databases.

To obtain only relevant results, the search was restricted to the period after 2005. The reference lists of included articles were screened for any potentially missed papers.

A total of 2320 articles were identified by the search; PubMed returned 664 articles, Scopus returned 992 articles, and articles from additional sources were included (eg, 15 papers, which were identified from the *References* sections of the aforementioned papers and did not appear in the initial search results, were included). After excluding duplicates, a total of 1420 papers passed the initial filter and were subsequently screened based on their titles and abstracts, in terms of the objectives of this review. If a paper could not be clearly rejected or accepted based on its title or abstract because it did not match the study conditions, a full-text analysis was also carried out. To be considered as an appropriate paper, the following criteria had to be met: (1) the prototype or device was described and tested in water, (2) the device could be worn and interacted with (eg, displays, buttons, etc), (3) the device was fully functional without external equipment, and (4) the paper was written in English and published in a peer-reviewed academic source.

Wearables that could not function independently as a research object were excluded. These included individual sensors that did not function as an independent device and actuators, nonportable sensors, or systems that were not wearable devices [24-26].

**Figure 1:**
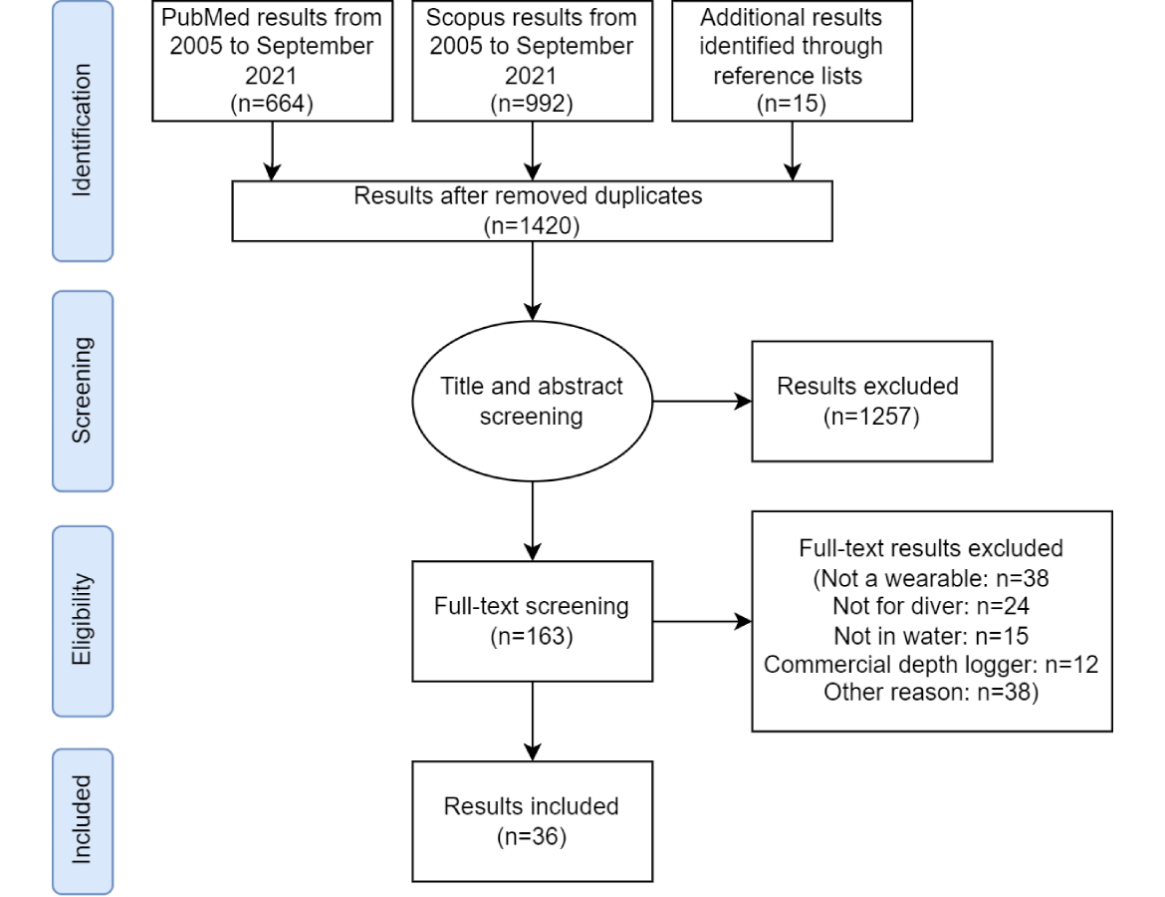
Flow diagram of study selection for wearable dive computers.

After a discussion involving all authors, we decided to include 171 studies in a full-text screening. A total of 41 articles were retained for a synthesis analysis, and 5 articles from this analysis were discarded at a later stage, since they did not fully meet the inclusion criteria. The individual steps that were carried out can be seen in Figure 1.

A spreadsheet table was created to present the data from the individual papers. Data extraction was performed by the first author. Articles in which the individual parameters of wearable devices were not clear were examined more closely by all authors, and a joint decision was made. There was no documented instance in which a final consensus was not achieved among the four authors. The data extracted from the articles that were included in this review were the types of articles, sources, titles, study topics, study samples, housing and sealing types, depth ratings, wearables’ locations on the body, implemented sensors and actuators, results, and other studies that used the same devices. In particular, the housing types and the tested depths were listed on a separate spreadsheet and displayed in a graphic. The applications of the studied wearables were compared with the respective preferred methods of wear; however, these data did not add any further value and were therefore discarded.

## Results

### Overview of Included Studies

Of the 36 studies retained in this review, 9 focused on underwater communication devices, 7 dealt primarily with the development of a head-up display (HUD), 2 dealt with underwater human-computer interaction possibilities, and the remaining 18 dealt with different kinds of safety devices. Theoretical measures were extracted from the wearable components’ specifications, if this information was available. Herein, the *maximum depth* is the depth at which a wearable was successfully used and tested underwater, and the *construction depth* is the theoretically possible depth, which was based on the designs and commercial specifications of the components reported in the corresponding publications.

### Use Cases That Wearables Cover, Besides a Depth Logger Dive Computer, and the Directions in Which They Are Developing

#### Safety Devices

##### Overview of Safety Devices

Half of the studies (18/36, 50%) dealt with divers' safety. This is nothing unusual, considering that when something dangerous happens underwater, it usually ends fatally [6]. The applied maximum test depths varied between 2.7 m and a theoretical 300 m. In this section and in the tables, both the tested and theoretical depth measures are analyzed.

Safety devices can be divided into and specified as further subcategories. The primary areas of application were vital signs (7 studies), the determination of a diver’s underwater position (7 studies), breathing detection (2 studies), and cognitive functions (2 studies). Although breathing detection is a part of the *vital signs* category, this has its own section due to its relevance and importance.

##### Vital Signs

As shown in Table 1, of the 7 studies on vital signs, 4 collected electrocardiogram values as the subject of the study. In doing so, depths ranging from 2.7 m up to 30 m were reached. All systems could be worn regardless of their location on the body or the need for external devices. Measuring oxygen saturation, blood pressure, and heart rate is particularly relevant for free divers.

**Table 1:** Studies covering vital signs.

Authors	Study topic	Maximum depth (construction depth), m	Sensors and actuators	Results
Tocco et al [27]	HR^a^, SV^b^, and CO^c^ during DA^d^	3 (90)	Miniaturized impedance cardiograph	No changes in HR, SV, and CO when compared with surface breathing and when immersed at the surface and at a 4-m depth
Tocco et al [28]	HR, SV, and CO	3 (90)	Impedance and ECG^e^ recorder	Bradycardia and decrements in SV and CO
Schuster et al [29]	Measuring body temperature (core and skin) and ECG monitoring	30 (N/A^f^)	ECG, temperature sensor, and Bluetooth sensor	Weak housing and problematic cables
Cibis et al [30]	Underwater monitoring of a diver’s ECG signal, including an alert system that warns the diver of predefined medical emergency situations	2.7 (N/A)	ECG sensor	Showed the good accuracy of the analysis system as well as the alert system
Kuch et al [31]	Wrist-mounted apnea dive computer for the continuous plethysmography monitoring of oxygen saturation and HR	11 (200)	Transcutaneous oxygen saturation, HR, plethysmography pulse waveform, depth, time, and temperature sensors	Continuous measurement of oxygen, HR, and plethysmography pulse waves for water temperature and depth was successful
Sieber et al [32]	Measurement of blood pressure underwater	10.5 (200)	Pressure sensor and sphygmomanometer	Accurate noninvasive measurement of blood pressure underwater
Di Pumpo et al [33]	Detecting peripheral oxygen saturation for an electronic closed-circuit rebreather diver	14 (N/A)	Pulse oximeter	Detecting pulse oximetry during an immersion makes diving with a rebreather safer

^a^HR: heart rate.

^b^SV: stroke volume.

^c^CO: cardiac output.

^d^DA: dynamic apnea.

^e^ECG: electrocardiogram.

^f^N/A: not applicable.

##### Breathing Detection

The detection of breathing has received too little attention in the literature so far but is of elementary importance for maintaining or increasing safety underwater. To know whether a scuba diver is drowning, it is helpful to know whether the diver is still breathing. Two different approaches were successfully tested (Table 2). A precision of over 97% was achieved with one device, which “read” the intermediate pressure signals on the scuba regulator and evaluated them via a built-in algorithm. In the second study, a textile sensor was attached to a diver's chest, which expanded accordingly while the diver breathed and thus provided different values. With this system, a breathing signal can be read independently of the scuba equipment. The studies achieved depths of 25 m [34] and 30 m [35], which are acceptable for recreational diving.

**Table 2:** Studies covering breathing detection.

Authors	Study topic	Maximum depth (construction depth), m	Sensors and actuators	Results
Altepe et al [34]	Breathing detection device	25 (100)	2 pressure sensors	Sensitivity as high as 97.5% for 16 dives after 13.9 hours of recording
Eun et al [35]	Enhance safety and collect biometric information	30 (N/A^a^)	MS5803-14BA pressure sensor (SparkFun Electronics) and respiratory sensors	Steps for entering the detailed menu should be shortened, and setting functions that were deemed to be unnecessary and dangerous (eg, rising speed warning alarm function) should be removed; diving computer usability obtained an overall average valuation of 84.7%

^a^N/A: not applicable.

##### Underwater Posture Determination of a Diver

The determination of a diver’s position under water has been pursued by many studies via multiple approaches (Table 3). In this review, a clear tendency toward a specific solution for recording the general position of a diver was not discernible, since the technologies described covered very different approaches. However, an automated buoyancy vest is particularly suitable for scuba and technical divers, as commercial products have already shown [36,37]. Additionally, a posture determination approach that is suitable for free divers can be carried out by means of a depth sensor and an inertial measurement unit (IMU) [38]. Other studies have also pursued the possibility of recording the unconscious behaviors of a diver via a camera (attached to the diver’s back) or have determined the position of a diver via a GPS or handheld sonar device.

**Table 3:** Studies covering underwater posture determination.

Authors	Study topic	Maximum depth (construction depth), m	Sensors and actuators	Results
Valenko et al [36]	Automatic buoyancy control	30 (80)	Pressure sensors (water and first-stage sensors), 3D accelerometer, and pneumatic valves	Initial correlations between the real dives and the simulated dive results were satisfactory.
Allotta et al [37]	Increase the safety of divers (aimed to detect the occurrence of too fast, possibly uncontrolled ascents of the diver)	300 (N/A^a^)	The *SARIS* system (pressure sensors were used but not described)	The proposed application of the *SARIS* system seems feasible.
Beluso et al [39]	Automatically collect and average pressure data	4.4 (30)	Pressure sensor, display, and magnetic induction switch	Sunlight and temperature affect the pressure sensor; therefore, misleading results for the depth were obtained.
Groh et al [38]	Underwater pose determination	N/A	3-axis accelerometer, 3-axis gyroscope, and camera	The system could analyze poses and fin kicks in real time.
Hirose et al [40]	Enhance the diving experience by recording users’ unconscious behaviors	N/A	Camera and wire transmission to the diver	The camera can capture the diver fully and even other diving members.
Kuch et al [41]	Accurate and affordable georeferencing for diver	N/A (300)	GPS, pressure sensor, and display	The authors reported an accuracy of <5 m.
McGrane et al [42]	Determine whether a handheld sonar device reduces the mean time for locating a missing diver	9 (N/A)	Mark Track sonar dive equipment (RJE International Inc)	The handheld sonar significantly reduces the mean duration for locating a missing diver.

^a^N/A: not applicable.

##### Cognitive Functions

To move in a strange and hostile environment, such as an underwater environment, intact cognitive functions are required. Although this topic is extremely relevant, only 2 systems that could function independently were identified (Table 4). In one study [43], the effects of cold water and cognitive impairment were recorded, with a significant increase of 111.7% in critical flicker fusion frequency values. The other study [44] could only determine a reduced performance at a depth of 20 m when processing the Stroop test; at a depth of 5 m or on land, no changes were found.

**Table 4:** Studies covering cognitive functions.

Authors	Study topic	Maximum depth, m	Sensors and actuators	Results
Piispanen et al [43]	CFFF^a^ test	45	Display and flickering light-emitting diode light	Increase of 111.7% in CFFF values when compared to those in predives; skin temperature dropped by 0.48 °C
Steinberg and Doppelmayr [44]	Stroop test, Number/Letter test, 2-back test, and a simple reaction time test	20	Heart rate sensor and pressure tank air stored with Galileo Sol (Johnson Outdoors Inc)	Several findings and results

^a^CFFF: critical flicker fusion frequency.

#### Head-Mounted Display Devices

HUDs are difficult to manufacture for mainstream use and are difficult to design in an appealing way, as the aesthetics are primarily determined by the manufacturer based on functionality. As a result, only large manufacturers of electronic devices could claim this market for themselves; they developed HUDs for everyday use, such as Microsoft HoloLens, Google Glass, or Intel Vaunt, but even the production of these HUDs has partially stopped for the time being due to their lack of acceptance as mainstream devices [45,46], even though they are currently offered to help people carry out work activities. However, since functionality is clearly in the foreground of diving and a diving mask or a full-face mask is used in diving anyway, the acceptance of HUDs among divers is substantially higher. As can be seen in Table 5, there are relatively many studies devoted to a HUD for divers. Due to the extensive integration of HUD technology into the masks themselves and the associated balanced pressure, most of the HUDs for diving can withstand a significantly greater depth or pressure without any problems. This is of particular benefit to technical divers, as their diving environment is the most likely to require HUD use. Of the 7 studies on HUDs, only 2 used a see-through mounted HUD. Only 1 of the 2 HUDs can be attached to a conventional mask and thus can be potentially used in free diving. Since HUDs are often integrated into full-face masks, these HUDs are not subject to the classic challenges of wearable devices and can instead be placed in masks without waterproof housing and without direct seals. As a result, these HUDs can reach significantly greater depths than those reached by HUDs mounted outside of masks.

**Table 5:** Studies covering head-mounted display devices.

Authors	Type of HUD^a^	Mounting	Dive mode	Maximum depth (construction depth), m	Sensors and actuators
Koss and Sieber [47]	Not see- through	Mounted outside of a mask	Rebreather diving	300 (N/A^b^)	HUD, 3 pO_2_^c^ sensors, depth sensor, time sensor, and decompression obligation sensor
Sieber et al [48]	Not see- through	Mounted inside of a full-face mask	Scuba diving, surface-supplied gas diving, and rebreather diving	45 (100)	Full-color display, depth sensor, tilt-compensated compass, and tank pressure sensor
Gallagher et al [49]	Not see- through	Mounted outside of a mask	Military combat diving	N/A	Microdisplay, optical lens, electronic compass, depth sensor, microprocessor, associated electronics, and battery
Gallagher and Manley [50]	See-through	Mounted inside of a mask	Scuba diving, surface-supplied gas diving, and rebreather diving	9 (N/A)	Depth sensor, compass, light-emitting diodes, and HUD
Manley et al [51]	See-through	Diving helmet	Military combat diving	12 (N/A)	HUD
Koss and Sieber [52]	Not see- through	Mounted outside of a mask	All (copies the dive computer screen)	95 (300)	Bluetooth sensor, pressure sensor, display, buttons, and pO_2_ sensor
Sieber et al [53]	Not see- through	Mounted outside of a mask	Rebreather diving	130 (N/A)	Infrared receiver, 3-axis IMU^d^, pressure sensor, tank pressure sensors, galvanic pO_2_ sensors, display, and buttons

^a^HUD: head-up display.

^b^N/A: not applicable.

^c^pO_2_: partial pressure of oxygen.

^d^IMU: inertial measurement unit.

#### Underwater Communication Devices

Underwater communication is the most important aspect of a wearable for the Internet of Underwater Things (IoUT) [12]. Without a wireless connection between each device and to the internet, the IoUT would not be able to establish itself. This is why wireless connectivity is of particular importance to the future development and establishment of the IoUT.

Due to the complexities and sizes of the modems for wireless underwater transmission, there is so far only a handful that has been successfully implemented and tested in a wearable.

As seen in Table 6, two such wearables fall back on a 2-part solution in which the transmitter or receiver is attached to the back, and the diver simply connects a wearable to the device on their back [54,55]. Furthermore, apart from those in a study by Bube et al [56], none of the wearables can reach a range of more than 20 m, which is not sufficient for meaningful use. Additionally, the data rate also decreases considerably as the communication range increases.

**Table 6:** Studies covering underwater communication devices.

Authors	Communication technology	Data rate	Maximum range, m	Power consumption, W	Depth, m	Sensors and actuators
Hussein et al [57]	Light	N/A^a^	A few meters	N/A	1	N/A
Kohlsdorf et al [58]	Acoustic	N/A	18 (direct positioning)	>5 and <10	N/A	Hydrophones, speaker, keyboard, and display
Cardia et al [54]	Acoustic	N/A	N/A	N/A	N/A	N/A
Anjangi et al [55]	Acoustic	N/A	>50	N/A	6.6	Beacon (GPS), acoustic communication, MS5837-30BA sensor (TE Connectivity), and pressure transducer
Chen et al [59]	Optical	500 kB/s	20	<10	30	Camera, photoelectric sensor, audio acquisition, and display
Katzschmann et al [60]	Acoustic	20 bytes/s	15	N/A	18	Acoustic transducer, depth sensor, and IMU^b^
Kuch et al [61]	GPS/GSM^c^ cable	N/A	N/A	N/A	16	Pressure sensor, tank pressure sensor, GPS/GSM, and display
Bube et al [56]	Acoustic	64 bytes/s	200	2.6	250	Pressure sensor, RTC^d^, acoustic modem, temperature sensor, heartbeat sensor, and display
Navea and Claveria [62]	Light	4 kB/s	7	N/A	1.5	Light sensors, earphones, and phototransistors

^a^N/A: not applicable.

^b^IMU: inertial measurement unit.

^c^GSM: Global System for Mobile Communications.

^d^RTC: real-time clock.

#### Human-Computer Interaction Approaches

The interaction with a conventional dive computer usually takes place via various buttons that are sealed against the ambient pressure under water. Although there is great potential for improvement in this area, only 2 of the papers dealt with the topic of interaction, as seen in Table 7. For this purpose, both the implementation of a touch screen that was insensitive to water pressure and the implementation of interaction via tilting the device for input were tested. For both variants, the advantage over button-based interaction stands out. Furthermore, there is no need for a physical connection to the outside of the housing, which always represents a potential weak point.

In both studies, good results were achieved under all conditions, which makes conducting further tests in this direction appear sensible. A comparison between the two interaction options and those for interacting with a classic dive computer via buttons makes sense.

**Table 7:** Studies covering human-computer interaction approaches.

Authors	Interaction type	Mounting type	Maximum depth, m	Sensors and actuators
Lee and Jun [63]	Touch screen	Wrist	50	Temperature, water pressure, and direction sensors
Čejka et al [64]	Tilting for underwater typing	Handheld	5	Samsung S8 sensors

### The Depths at Which the Wearables Were Used and the Most Forward-looking Seals

Particular attention should be paid to the implementation of housing in a large number of different studies, as this is currently one of the greatest hurdles for the development of new and innovative ideas for the IoUT. As can be seen in Table 8, almost every housing type was used for different study designs. Nevertheless, a clear tendency in the choices of the primarily used housing types was seen. The most commonly reported housing type was a polymer or polymethylmethacrylate (transparent thermoplastic plastic) housing (studies: 15/36, 42%). This was followed by aluminum cases (studies: 7/36, 19%) and commercial smartphone, tablet, or bag cases (studies: 9/36, 25%). There were also instances of devices being sealed either in a diving helmet (studies: 3/36, 8%) or with a potting compound (studies: 3/36, 8%). Only 1 of the 36 (2%) studies used tempered glass for the housing. In 2 of the 36 (5%) studies, no information on the housing was given.

**Table 8:** Housing and sealing comparison.

Housing and sealing type and study topic				Tested depth (construction depth), m
**Aluminum case**				
	**Communication**			
		Kohlsdorf et al [58]	N/A^a^	
		Kuch et al [61]	16 (N/A)	
	**Head-up display**			
		Sieber et al [48]^b^	45 (N/A)	
	**Safety device**			
		Valenko et al [36]^b^	30 (80)	
		Altepe et al [34]	25 (100)	
		Kuch et al [41]	N/A (300)	
		Di Pumpo et al [33]^b^	14 (N/A)	
**Commercial smartphone housing, tablet housing, or bag**				
	**Communication**			
		Hussein et al [57]	1 (N/A)	
		Cardia et al [54]	N/A	
		Anjangi et al [55]	6.6 (N/A)	
	**Interaction**			
		Čejka et al [64]	5 (N/A)	
	**Safety device**			
		Beluso et al [39]	4.4 (30)	
		Steinberg and Doppelmayr [44]	20 (N/A)	
		Groh et al [38]	N/A	
		Schuster et al [29]	30 (N/A)	
		Cibis et al [30]	2.7 (N/A)	
**Diving helmet or mask**				
	**Communication**			
		Chen et al [59]	30 (N/A)	
	**Head-up display**			
		Manley et al [51]	12 (N/A)	
	**Safety device**			
		Di Pumpo et al [33]^b^	14 (N/A)	
**Polymer or polymethylmethacrylate (Lexan, acryl, Plexiglas, etc)**				
	**Communication**			
		Navea and Claveria [62]	1.5 (N/A)	
		Katzschmann et al [60]	18 (N/A)	
		Bube et al [56]	250 (N/A)	
	**Head-up display**			
		Koss and Sieber [47]	300 (N/A)	
		Sieber et al [48]^b^	45 (100)	
		Gallagher et al [49]	N/A	
		Koss and Sieber [52]^b^	95 (300)	
	**Safety device**			
		Valenko et al [36]^b^	30 (80)	
		Kuch et al [31]	11 (200)	
		Tocco et al [27]	3 (90)	
		Sieber et al [32]	10.5 (200)	
		Piispanen et al [43]	45 (N/A)	
		Tocco et al [28]	3 (90)	
		Allotta et al [37]	300 (N/A)	
		Hirose et al [40]	N/A	
**Potting compound**				
	**Head-up display**			
		Gallagher and Manley [50]^c^	9 (N/A)	
		Koss and Sieber [52]^b^	95 (300)	
		Sieber et al [53]	130 (N/A)	
**Tempered glass**				
	**Interaction**			
		Lee and Jun [63]	50 (N/A)	
**Not specified**				
	**Safety device**			
		Eun et al [35]	30 (N/A)	
		McGrane et al [42]	9 (N/A)	

^a^N/A: not applicable.

^b^The device consists of 2 parts and is therefore listed in 2 housing and sealing categories.

^c^The depth is only 9 m because the potting compound was not applied to all components. Instead, the components had their own compartments, which is a problem with regard to sealing. The buttons were sealed with O-rings.

**Figure 2:**
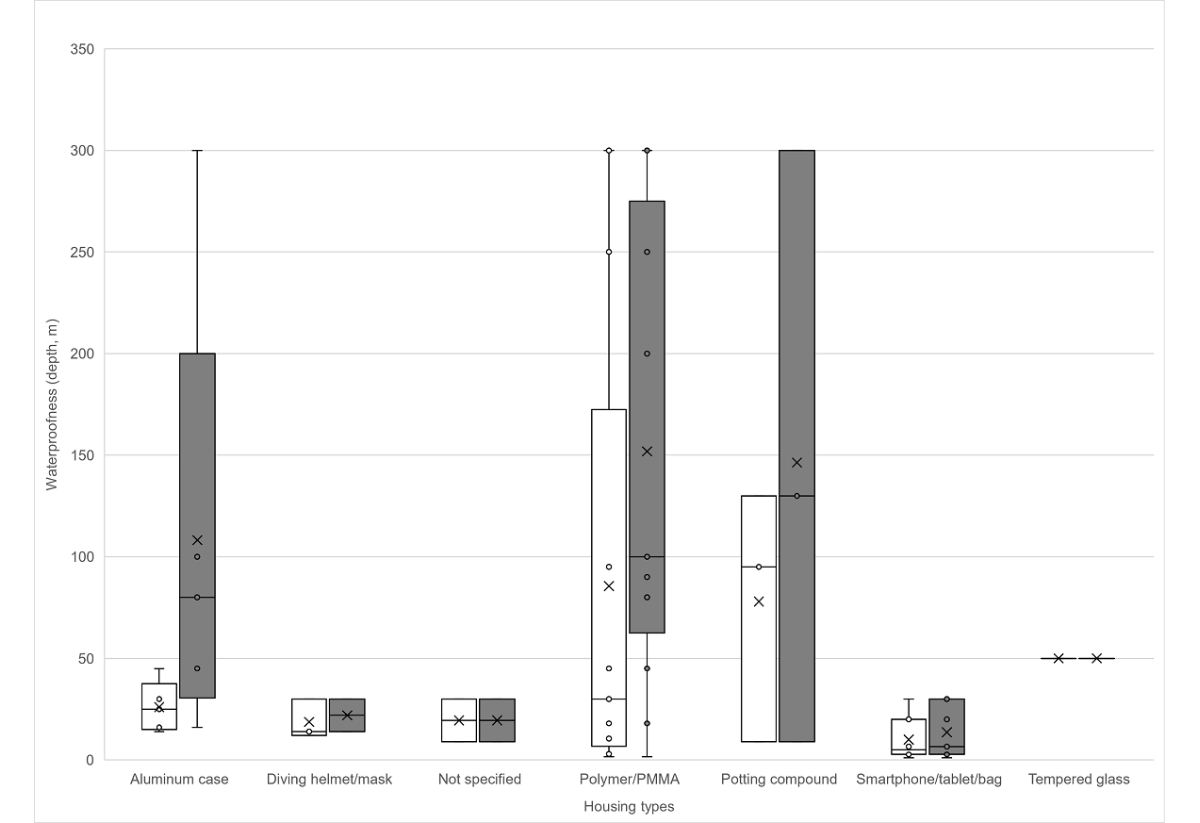
Housing type and waterproofness (ie, depth in meters). White bars indicate the tested and confirmed depths. Grey bars indicate the calculated or specified depths. PMMA: polymethylmethacrylate.

As expected, across all examined studies, the depth tested was well below the theoretical construction depth (Figure 2). Only the use of tempered glass was tested at the maximum specified depth. A direct comparison between housings that were made of a polymer or polymethylmethacrylate and housings that used a potting compound showed that the depths achieved by both housing types were approximately equivalent. By weighing the costs and benefits of a specific study that is to be carried out, a decision can be made between the two housing types. The most common primary cause cited against the use of a potting compound for housing was the difficulty in accessing the device (ie, for charging, programming, and interacting with the device) after pouring the compound [39]. If these challenges are overcome, the use of cast housings, including those used for underwater sensors, could prevail in the long term.

## Discussion

### Study Overview

Some of the insights that we gained from the reviewed studies were that many of the wearables examined were in the prototype stage or were only designed for a specific group of users. Safety-relevant devices received the greatest attention, and much of the technological progress in the development of underwater wearables can be attributed to their contributions to the field.

The most promising areas of development include underwater communication and human-computer interaction, as improvements in these areas will enable the entry of underwater wearables into a consumer market, which in turn can result in increased attention for such wearables in society and thus increased attention for science.

### Principal Results

#### Safety Devices

The collection and storage of vital values via underwater wearables received the most attention, since little is known about the medical background of diving, especially among divers and free divers. However, due to the advancing developments in this area within recent years, the use of such wearable devices has made it possible to achieve initial results. By linking previously developed and functioning safety devices with underwater communication devices, the almost real-time monitoring of a diver can take place within various disciplines in the future. Possible scenarios for the use of underwater wearables for vital sign monitoring include technical diving and free diving competitions, among others, as such wearables can be used to increase the safety conditions of these activities. By specifically measuring the vital parameters of free divers in all diving competitions, a significantly larger and more meaningful database can be accessed in the future. For subsequent developments in underwater safety wearables, predictive algorithms could be developed based on a vital sign database. This algorithm could be used to warn divers about critical conditions before they occur. This approach, as a concept, has already been presented but has not yet been tested in real life [65]. To achieve this however, the necessary prototypes must be significantly further developed, so that they can be used meaningfully outside of a scientific study, preferably as a finished consumer product.

As a result of the fact that divers can move freely in all 3 dimensions, contrary to land-based deployment, location and position determination received substantially higher levels of attention. In addition, since GPSs do not work when used in water, various localization options were investigated, but they showed weaknesses in various settings, such as in caves, in water with strong currents, and under great depths [41]. Location and position determination via an integrated IMU has shown promising results and should be further investigated. The almost real-time transmission of the underwater position of a diver also has many applications, such as the monitoring of individual students at a diving school; the early detection of dangers, such as currents and drifts; and, for free divers, the analysis of movement sequences by a trainer.

The use of breathing detection devices in combination with underwater communication devices also provides new possibilities. In addition, by using a developed respiratory sensor system, a further focus on the collection and evaluation of diaphragm contraction data from free divers can be achieved [35].

#### Underwater Communication

Underwater communication via multiple underwater devices and communication with people on the surface are fundamental pillars for an extensive network of wearables. However, the spread of this network is currently limited by 3 essential factors. These factors are the sizes, costs, and bandwidths of modules, which currently do not allow for any economic dissemination. Due to the constantly advancing developments in the IoUT and the underlying sensor networks, these modules will be useful in the future due to the scalability of wearables for underwater use. The uses of underwater communication wearables are diverse and can be expanded enormously.

By implementing the ahoi acoustic modem in a wearable, a significantly more compact and cheaper wearable for use underwater can be created in the future [66]. If the development of underwater wearables proceeds in the same manner as the development of mainstream wearables, underwater wearable development can focus on miniaturization and arrangement, which would benefit the current bulky modems that are used underwater [67]. By networking divers’ devices with each other and with the internet, the potential of these devices was often examined and shown in regard to the IoUT [7,9].

Through the further connection of wearables to other sensors on reefs, boats, or other underwater sites (eg, shipwrecks), safety-relevant information can also be transmitted to divers regardless of vital parameters, underwater locations, navigation limitations, or currents, and appropriate warnings (eg, the sudden appearance of dangers, changes in current direction, etc) can be given to avoid accidents. An overview of the variety of safety options was provided by Jahanbakht et al [12].

As soon as underwater communication wearables can be made to be cheaper and more compact, other subaspects, such as the collection of vital parameter data, will automatically improve. The first promising step toward a more cost-effective and compact device with an acceptable communication range has already been presented [56]. A wearable device with data transmission capabilities can be manufactured commercially through consistent and further developments that are based on previous approaches. However, free divers’ willingness to spend more money on unique products has yet to be investigated.

Of note, since GPSs do not work underwater, a different approach is required with regard to locating divers as well. If entry and exit points are recorded by a GPS, the underwater location of a diver can be determined via an IMU.

Algorithms that can recognize whether a diver is in danger based on movement data can also be used to make enormous progress toward locating divers and significantly increasing their safety. This approach has already been described in great detail for land-based use cases [68,69]. Vinetti et al [14] concluded that the monitoring and transmission of oxygen levels, as well as related feedback, and the most effective economical swimming techniques will have the greatest impact in the future. Ours is the first review to consider both the data monitoring aspects and data transmission aspects of underwater wearables. If these aspects are optimized, we believe that further developments for the IoUT will be made in the future and will have the greatest impact on the underwater world.

#### Human-Computer Interaction

Because the focus of the development of dive computers has so far been almost exclusively on computer science studies, it is not surprising that the human-computer interaction aspect has only been researched very rudimentarily so far. As a result, the number of studies that have been carried out on this subject has been very limited. However, the two identified interaction approaches showed a clear trend and the associated need for further investigations. A trend toward a design without weak points and connections to external components can be seen in the literature. In one study [63], interaction via the classic touch screen was chosen as the interaction method, and the other study [64] opted for interaction via tilting the device. As soon as the commercialization of underwater data communication becomes better established, as with mainstream wearables, human-computer interaction with regard to underwater wearables could gain importance and attention in the next years [70]. However, it is possible that insufficient attention is paid to this subject, which has been the case for the mainstream wearable market in recent years [67].

In the context of underwater wearables, whether interactions that do not require external components turn out to be better or more useful than interactions that do require such components (ie, buttons) should be further pursued and investigated. It may well be that the general paradigms of usability and user experience that are applied to land-based devices cannot be applied to underwater devices to the same extent. So far however, no studies have been carried out in this direction to our knowledge. Nevertheless, as soon as underwater data communication is offered as a commercial function of dive computers, the human-computer interaction aspect of underwater wearables could receive increased attention, since improvements in this aspect would result in completely new methods of interaction and extended functionalities that could address the interaction needs of users.

#### Housing and Sealing

Several lines of evidence suggest that the sealings used to protect against underwater environmental influences will continue to distinguish the development of wearables for underwater use from the development of wearables for land-based use in the future. As far as our review shows, no study has dealt with this subject before. Per the data we gathered on housing and sealing types and their respective achieved or projected depths, we assume that polymer or polymethylmethacrylate housings and cast housings will continue to be the primarily used housing types in the future [47]. Cast housings can be used to eliminate various problems, such as heat generation, programmability issues, chargeability issues, and user-friendliness issues. Furthermore, the next step should be a careful study of the relationships among cost, aesthetics, design, and achievable depth, which have direct implications for various seals. These are particularly important with regard to whether a completely casted housing is accepted for a commercial product. Even though the individual reasons were not mentioned, a completely encapsulated housing was only used for 3 HUDs [50,52,53]. Therefore, no clear trend can be identified in this area. The reasons for not using such housings could have been poor aesthetics, poor maintainability, and dissatisfaction with the elimination of the heat generated by a device. The introduction of different colored epoxy adhesives, along with light-emitting diodes and a special form of dive computer, could certainly appeal to the market.

### Comparison With Prior Work

To date, many reviews on underwater wearables have focused exclusively on the collection and evaluation of physiological and psychological parameters during diving as the primary research objective. These studies generally focused on available sensors that can be adapted for underwater use [13], dealt exclusively with the sensors and not with the entire wearable [14], or only dealt with devices that measured physiological parameters [15].

Apart from the fact that some of the papers we reviewed were published a few years ago, they mainly dealt with safety-related aspects in the field of diving. This trend was repeatedly confirmed in this review, since half of the reviewed studies (18/36, 50%) examined wearable devices that were related to safety. Furthermore, the safety-related wearable devices identified in this review were largely used in conjunction with devices from other studies. However, these other studies were excluded because the devices they used could not be used as a wearable, per the criteria outlined in the *Methods* section, or because they were published after the review period.

Ours is the first review on underwater devices, and we provide a first look at their potential and the challenges associated with their development. So far, the development areas of underwater communication and human-computer interaction for divers have not received any real attention. Furthermore, this is the first review to summarize the available diving devices that can be considered scientifically tested wearable prototypes. Commercial dive computers themselves have already been studied in terms of various parameters, such as precision in measuring depth and ergonomic performance [18,19]. A review of modern dive computers and a comparison of 47 dive computer models, which involved a comparison of their specifications, were carried out before [16,17]. By gathering data on the housing types of wearable devices along with the maximum tested depths and the theoretically calculated depths at which the devices remained functional, we were also able to show the tendencies in this area, which have not been shown before, as far as we know.

### Conclusions

This scoping review shows a first comprehensive insight into the various subaspects of developed prototypes of wearable devices for underwater use. The possibilities and challenges of the reviewed technologies were considered and evaluated separately. In addition to the well-covered field of safety devices that relate to the collection of vital sign data from divers, other areas such as underwater communication between divers, as well as topics such as human-computer interaction and specialized wearables for divers, were covered for the first time. Recent research has shown that underwater communication has the most significant influence on future developments. In contrast, human-computer interaction has so far received far too little consideration. This is particularly surprising because the conditions under water are different from those on the surface. A scientific summary and overview of the housings and seals used among devices for scientific purposes should be considered in the future and on a larger scale.

In their current state, none of the devices reviewed in this study can prompt the further development of underwater wearables. The greatest future impacts will result from a combination of all of the aspects mentioned herein, with a special focus on safety and communication. The trends seen with mainstream wearables can thus be seen with underwater wearables as well, which focus primarily on sensor design, communication protocols, and data processing and analysis [67]. If these trends continue, underwater safety devices could be used to communicate with other divers and stations in the IoUT and, if necessary, immediately carry out an action. This could, for example, significantly shorten and optimize a rescue chain in an emergency. By focusing research on wearable devices for underwater use and further developing them into consumer products, such underwater networking could also be used for subareas other than safety measures or the collection of human physiology data [13]. The possible application scenarios could include the maintenance and repair of underwater structures, such as bridges or drilling platforms; the collection and evaluation of data from animals by using sensor materials; or the broad-based collection of data on submarine environments by using wearable devices underwater.

## Abbreviations

HUD: head-up display
IMU: inertial measurement unit
IoUT: Internet of Underwater Things
PRISMA: Preferred Reporting Items for Systematic Reviews and Meta-Analyses
PRISMA-ScR: Preferred Reporting Items for Systematic Reviews and Meta-Analyses extension for Scoping Reviews
